# Associations of Chronic Inflammation, Insulin Resistance, and Severe Obesity With Mortality, Myocardial Infarction, Cancer, and Chronic Pulmonary Disease

**DOI:** 10.1001/jamanetworkopen.2019.10456

**Published:** 2019-08-30

**Authors:** Natasha Wiebe, Peter Stenvinkel, Marcello Tonelli

**Affiliations:** 1 Department of Medicine, University of Alberta, Edmonton, Alberta, Canada; 2 Department of Renal Medicine, Karolinska University Hospital, Stockholm, Sweden; 3 Department of Medicine, University of Calgary, Calgary, Alberta, Canada

## Abstract

**Importance:**

Chronic inflammation and insulin resistance often accompany severe obesity, and all are associated with disease risk.

**Objective:**

To examine how the association of severe obesity with adverse outcomes may be modified by the presence of systemic inflammation and/or insulin resistance.

**Design, Setting, and Participants:**

This population-based, retrospective cohort study included all residents of Alberta, Canada, aged 18 years and older with at least 1 procedure to ascertain severe obesity and measures of C-reactive protein, fasting glucose, triglyceride, and high-density lipoprotein cholesterol levels. Participants were observed from April 2003 to March 2017, and data analysis was conducted from June 2018 to December 2018.

**Exposures:**

Severe obesity (body mass index ≥35 or ≥40 after January 1, 2017, as indicated with a procedure-fee modifier), chronic inflammation (all measures of C-reactive protein >10 mg/L), and a surrogate measure of insulin resistance.

**Main Outcomes and Measures:**

All-cause death, first acute myocardial infarction during follow-up, first cancer diagnosis during follow-up, and new chronic pulmonary disease.

**Results:**

Among 420 636 participants, the median age was 45 years (interquartile range, 34-56 years; range, 18-97 years), 157 799 (37.5%) were male, 185 782 (44.2%) had insulin resistance, 71 987 (17.1%) had severe obesity, and 10 770 (2.6%) had inflammation. In women with chronic inflammation, the presence of severe obesity was associated with a lower mortality risk (hazard ratio [HR], 0.75; 95% CI, 0.65-0.86), but there was no difference in risk in men with inflammation (HR, 0.89; 95% CI, 0.78-1.02). In contrast, the presence of severe obesity was associated with a higher mortality risk in men without inflammation (HR, 1.20; 95% CI, 1.13-1.26), but there was no difference in risk in women without inflammation (HR, 1.00; 95% CI, 0.95-1.06). For myocardial infarction, severe obesity was associated with increased risk in both women and men without inflammation (women: HR, 1.26; 95% CI, 1.17-1.36; men: HR, 1.35; 95% CI, 1.27-1.43) but not in women and men with inflammation (women: HR, 0.85; 95% CI, 0.67-1.07; men: HR, 0.90; 95% CI, 0.71-1.14). Severe obesity was associated with increased risk in women and men, irrespective of chronic inflammation, for new chronic pulmonary disease (women with inflammation: HR, 1.34; 95% CI, 1.23-1.46; women without inflammation: HR, 1.58; 95% CI, 1.54-1.62; men with inflammation: HR, 1.41; 95% CI, 1.29-1.54; men without inflammation: HR, 1.65; 95%, CI, 1.60-1.71) and cancer (women with inflammation: HR, 1.16; 95% CI, 1.03-1.30; women without inflammation, HR, 1.32; 95% CI, 1.28-1.36; men with inflammation: HR, 1.17; 95% CI, 1.04-1.32; men without inflammation: HR, 1.33; 95% CI, 1.28-1.39). Similar to chronic inflammation, severe obesity was not always associated with higher risk in participants with insulin resistance.

**Conclusions and Relevance:**

The findings suggest that severe obesity with systemic inflammation is associated with a different prognosis than severe obesity without inflammation.

## Key Points

**Question:**

Is the association of severe obesity with adverse outcomes modified by the presence or absence of systemic inflammation?

**Findings:**

In this cohort study of 420 636 Canadian adults, severe obesity was associated with a lower mortality risk in women with chronic inflammation, but there was no difference in risk in men with inflammation. In contrast, severe obesity was associated with a higher mortality risk in men without inflammation, but not with mortality risk in women without inflammation.

**Meaning:**

Severe obesity in the presence of systemic inflammation is associated with a different prognosis compared with severe obesity in the absence of inflammation.

## Teaser

This population-based cohort study among Canadian adults examines how the associations of severe obesity with adverse outcomes may be modified by the presence of systemic inflammation and/or insulin resistance.

## Introduction

The prevalence of obesity in Western countries has increased in parallel with the prevalence of biochemical parameters suggesting chronic inflammation.^1^ Consistent with this, individuals with obesity have higher mean levels of inflammatory biomarkers.^2^ Although the explanation for this observation is difficult to ascertain, there are multiple inflammatory conditions that are associated with obesity,^3^ including type 2 diabetes, heart disease, chronic kidney disease, cancer, asthma, gastroesophageal reflux disease, psoriasis,^4^ and small intestine bacterial overgrowth.^5^

In energy-restricted dietary trials, weight loss and maintenance can be projected from baseline levels of insulin and inflammation, with participants who have the highest weight loss consistently having lower levels of baseline insulin and inflammatory biomarkers.^6^ Longitudinal studies,^7,8,9,10,11,12,13,14^ drug trials,^15,16,17^ and bariatric surgery^18^ have shown that weight changes are preceded by changes in insulin and inflammation. Although the common view^19^ is that obesity causes insulin resistance (IR) and inflammation, an alternative explanation is that elevated insulin and inflammation induce weight gain in the presence of disease to the host’s advantage. If confirmed, this might help to explain the so-called survival paradox whereby overweight and obesity are associated with increased life expectancy in populations with chronic disease as well as those older than 60 years.^20^

This study examined the joint associations of chronic inflammation, IR, and severe obesity with the risks of a set of adverse clinical outcomes, including all-cause death, incident acute myocardial infarction (AMI), incident cancer diagnosis, and new chronic pulmonary disease. Our goal was to examine how the association of severe obesity with adverse outcomes was modified by the presence or absence of systemic inflammation and/or IR. We examined these associations separately for women and men because there is some evidence that sex may modify the survival benefit attributed to obesity in cardiac conditions.^21,22^

## Methods

This retrospective, population-based cohort study is reported according to the Strengthening the Reporting of Observational Studies in Epidemiology (STROBE) reporting guideline.^23^ The institutional review boards at the University of Alberta and University of Calgary approved this study and waived the requirement for participants to provide consent because of the large sample size. The analysis was conducted between June 2018 and December 2018.

### Data Sources and Cohort

We used the Alberta Kidney Disease Network database, which incorporates data from the provincial health ministry Alberta Health (AH), specifically from their registry, physician claims, hospitalizations, and ambulatory care utilization, and from the clinical laboratories in Alberta, Canada. This database has been widely used^24,25,26^ because of its population-based coverage of a geographically defined area; its data include demographic characteristics, health services utilization, and clinical outcomes. Additional information on the database is available elsewhere, including the validation of selected data elements and the standardization and calibration of serum creatinine assays.^27^ All adults registered with AH were included in the database; all Alberta residents are eligible for insurance coverage from AH and more than 99% participate in coverage. The database was used to assemble a cohort of adults who resided in Alberta, had a minimum of 2 C-reactive protein (CRP) measures over a period of longer than 1 year, and had a procedure and values for fasting glucose and cholesterol levels. We observed participants from April 1, 2003, or registration with AH, whichever was later (baseline), until death, out-migration, or study end (March 2017), whichever was earliest.

### Inflammation, IR, and Severe Obesity

We considered chronic systemic inflammation to be present if all values of CRP were higher than 10 mg/L^28^ (to convert to nanomoles per liter, multiply by 9.524) over a period of longer than 1 year, with at least 2 values to exclude acute infections. The threshold of 10 mg/L was chosen to exclude participants with elevations of CRP owing to vascular disease, as opposed to higher thresholds that might include only those with infection, an autoimmune condition, or a cancer.^28^ Because fasting insulin level was not available in our data, we used commonly measured parameters^29^ for our surrogate for IR rather than more robust indices such as the Homeostatic Model Assessment of Insulin Resistance or the Quantitative Insulin Sensitivity Check Index.^30^ Our surrogate for IR was defined as at least 2 of the following fasting parameters: triglyceride levels of 142 mg/dL or higher (to convert to millimoles per liter, multiply by 0.0113), glucose levels of 108 mg/dL or higher (to convert to millimoles per liter, multiply by 0.0555), or high-density lipoprotein cholesterol (HDL-C) levels of 39 mg/dL or lower (to convert to millimoles per liter, multiply by 0.0259), determined from available values closest to baseline. We also considered the metabolic index,^29^ which combines these 3 parameters ([triglyceride level × glucose level] / HDL-C level squared) and defines values 7 and higher as indicating IR. Lookback for laboratory values extended to May 2002 where records were available.

We used health service codes from the physician claims data to identify procedures performed by a physician. From July 1, 2007, to December 31, 2016, AH has supplemented physician procedure fees by 25% when surgical, obstetrical, bronchoscopic, or endoscopic procedures are performed on patients with body mass index (BMI, calculated as weight in kilograms divided by height in meters squared) of 35 or higher.^31^ Starting January 1, 2017, the threshold for the supplemental fee modifier was increased to BMI of 40 or higher. Full details of these procedures have been published previously.^32^ Thus, any participant undergoing any procedure (at any point after July 1, 2007) was included in the cohort. Participants were classified as having severe obesity if a claim for any procedure included a fee-modification code indicating that severe obesity was present, and otherwise, they were classified as not having severe obesity.

### Comorbidities

Comorbidities were defined using a previously published framework with 29 validated algorithms as applied to Canadian physician claims data, each of which had positive predictive values of 70% or higher compared with a criterion-standard measure, such as medical record review.^33^ Comorbidities included alcohol use disorder, asthma, atrial fibrillation, lymphoma, nonmetastatic cancer (breast, cervical, colorectal, pulmonary, and prostate cancer), metastatic cancer, chronic heart failure, chronic pain, chronic obstructive pulmonary disease, chronic hepatitis B, cirrhosis, severe constipation, dementia, depression, diabetes, epilepsy, hypertension, hypothyroidism, inflammatory bowel disease, irritable bowel syndrome, multiple sclerosis, AMI, Parkinson disease, peptic ulcer disease, peripheral artery disease, psoriasis, rheumatoid arthritis, schizophrenia, and stroke or transient ischemic attack. Each participant was classified with respect to the presence or absence of these 29 chronic conditions before or at baseline (lookback extended as far as April 1994 where records were available).^34^ Detailed methods for classifying comorbidity status and the specific algorithms used are found elsewhere.^33^ We also considered chronic kidney disease as a 30th condition, which was defined by mean annual estimated glomerular filtration rate below 60 mL/min/1.73 m^2^ or the presence of albuminuria (albumin-to-creatinine ratio, ≥30 mg/g; protein-to-creatinine ratio, ≥150 mg/g; or dipstick proteinuria, ≥trace).

### Outcomes

Clinical outcomes were all-cause death, first AMI during follow-up,^33,35^ first cancer diagnosis during follow-up, and new chronic pulmonary disease^31,36,37^ (eg, chronic obstructive pulmonary disease, bronchitis, pneumoconiosis, or asthma in those without prior chronic pulmonary disease). Cancers included solid tumors (breast, cervical, colorectal, lung, or prostate cancer^38^), lymphoma,^36^ and metastatic cancer of any origin.^36^

### Statistical Analysis

We performed analyses with Stata MP version 15.1 (StataCorp) and reported baseline descriptive statistics as counts and percentages or medians and interquartile ranges (IQRs) as appropriate. Differences were tested using the χ^2^ or Kruskal-Wallis tests. Unadjusted risk ratios (RRs) were calculated to measure the associations of the 3 main exposures with outcomes. We used age-adjusted and fully adjusted Cox regression to determine the associations of the 8 permuted groups of inflammation, IR, and severe obesity as well as sex with the clinical outcomes. Age was categorized as follows: 18 to 39 years, 40 to 64 years, 65 to 79 years, and 80 years or older. In the fully adjusted models, we additionally adjusted for social assistance and 30 comorbidities. All 2-way interactions between inflammation, IR, severe obesity, and sex were included. Sex was included as it may modify the association of severe obesity with survival. Participants with chronic pulmonary disease before baseline were excluded from analyses examining time to new pulmonary disease. In a sensitivity analysis, we substituted metabolic index of 7 or higher^29^ for our surrogate of IR. We determined that the proportional hazard assumption was satisfied by examining plots of the log-negative-log of within-group survivorship probabilities vs log-time. The threshold for statistical significance was set at *P* < .05, and all tests were 2-tailed.

## Results

### Characteristics of Study Participants

Participant flow is shown in the eFigure in the Supplement. Overall, 4 089 586 Alberta residents (90.7%) were excluded because they did not have the required bloodwork and/or a procedure. This excluded set of people were both older and younger, contained proportionally more men, and had less frequent comorbidity except for dementia (eTable 1 in the Supplement).

Among 420 636 participants, the median age was 45 years (IQR, 34-56 years; range, 18-97 years), 157 799 (37.5%) were male, 185 782 (44.2%) had IR, 71 987 (17.1%) had severe obesity, and 10 770 (2.6%) had inflammation. Participants were observed for a median of 14 years (IQR, 14-14 years; range, 1 month to 14 years). There were 19 351 deaths (4.6%); 58 335 participants (13.9%) developed a new chronic pulmonary disease (26 155 [6.2%] had a chronic pulmonary disease at baseline), 40 837 (9.7%) had incident solid malignant neoplasm or lymphoma during follow-up, and 12 030 (2.9%) had at least 1 AMI during follow-up.

Table 1 summarizes participants’ demographic and clinical characteristics by chronic inflammation, IR, and severe obesity. Severe obesity was almost 3-fold more likely among participants with inflammation (RR, 2.76; 95% CI, 2.70-2.82) as compared with those without inflammation. Insulin resistance was nearly twice as likely among participants with inflammation (RR, 1.84; 95% CI, 1.77-1.91), and severe obesity was about 1.5-fold more likely among participants with IR (RR, 1.55; 95% CI, 1.54-1.56). While severe obesity and IR were associated with inflammation, few participants with severe obesity or IR had inflammation (6.8% and 3.4%, respectively).

**Table 1.** Demographic and Clinical Characteristics by Inflammation, Insulin Resistance, and Severe Obesity

Characteristic	Inflammation, No. (%)^a^	No Inflammation, No. (%)	*P* Value	Insulin Resistance, No. (%)^b^	No Insulin Resistance, No. (%)	*P* Value	Severe Obesity, No. (%)^c^	No Severe Obesity, No. (%)	*P* Value
No.	10 770 (2.6)	409 866 (97.4)	NA	185 782 (44.2)	234 854 (55.8)	NA	71 987 (17.1)	348 649 (82.9)	NA
Age, y									
Median (IQR)	44 (32-56)	45 (34-56)	<.001	48 (38-59)	43 (32-53)	<.001	46 (34-56)	45 (35-56)	<.001
18-39	4246 (39.4)	144 421 (35.2)	<.001	50 807 (27.3)	97 860 (41.7)	<.001	25 341 (35.2)	123 326 (35.4)	<.001
40-64	5111 (47.5)	216 818 (52.9)		108 137 (58.2)	113 792 (48.5)		40 213 (55.9)	181 716 (52.1)	
65-79	1269 (11.8)	45 377 (11.1)		25 240 (13.6)	21 406 (9.1)		6302 (8.8)	40 344 (11.6)	
≥80	144 (1.3)	3250 (0.8)		1598 (0.9)	1796 (0.8)		131 (0.2)	3263 (0.9)	
Women	7693 (71.4)	255 144 (62.3)	<.001	90 736 (48.8)	172 101 (73.3)	<.001	50 081 (69.6)	212 756 (61.0)	<.001
Receiving social assistance	557 (5.2)	11 678 (2.8)	<.001	6343 (3.4)	5892 (2.5)	<.001	2876 (4.0)	9359 (2.7)	<.001
Laboratory results, median (IQR)									
CRP level, mg/L	19.8 (15.2-27.9)	2.9 (1.3-6.4)	<.001	3.7 (1.8-8.7)	2.5 (1.1-5.6)	<.001	5.7 (2.9-11.4)	2.7 (1.1-5.9)	<.001
HDL-C level, mg/dL	42 (36-54)	49 (38-61)	<.001	37 (35-48)	56 (48-66)	<.001	41 (36-54)	50 (38-62)	<.001
Glucose level, mg/dL	105 (88-115)	94 (86-110)	<.001	110 (97-119)	90 (85-95)	<.001	108 (90-115)	94 (86-108)	<.001
Triglyceride level, mg/dL	160 (11-204)	152 (88-191)	<.001	181 (154-232)	103 (70-156)	<.001	167 (142-214)	148 (84-187)	<.001
Morbidities									
Median (IQR), No.	1 (0-2)	0 (0-1)	<.001	1 (0-2)	0 (0-1)	<.001	1 (0-2)	0 (0-1)	<.001
Chronic pain	2187 (20.3)	82 094 (20.0)	.48	40 851 (22.0)	43 430 (18.5)	<.001	18 428 (25.6)	65 853 (18.9)	<.001
Hypertension	2594 (24.1)	73 294 (17.9)	<.001	49 045 (26.4)	26 843 (11.4)	<.001	19 730 (27.4)	56 158 (16.1)	<.001
Depression	1431 (13.3)	45 642 (11.1)	<.001	21 985 (11.8)	25 088 (10.7)	<.001	10 746 (14.9)	36 327 (10.4)	<.001
Chronic pulmonary disease	1016 (9.4)	25 139 (6.1)	<.001	14 870 (8.0)	11 285 (4.8)	<.001	6490 (9.0)	19 665 (5.6)	<.001
Hypothyroidism	712 (6.6)	24 693 (6.0)	.01	12 025 (6.5)	13 380 (5.7)	<.001	5511 (7.7)	19 894 (5.7)	<.001
Diabetes	904 (8.4)	20 842 (5.1)	<.001	21 331 (11.5)	415 (0.2)	<.001	6916 (9.6)	14 830 (4.3)	<.001
Chronic kidney disease	480 (4.5)	10 411 (2.5)	<.001	7545 (4.1)	3346 (1.4)	<.001	2564 (3.6)	8327 (2.4)	<.001
Rheumatoid arthritis	315 (2.9)	9542 (2.3)	<.001	4479 (2.4)	5378 (2.3)	.01	1683 (2.3)	8174 (2.3)	.92
Asthma	448 (4.2)	9108 (2.2)	<.001	5169 (2.8)	4387 (1.9)	<.001	3091 (4.3)	6465 (1.9)	<.001
Stroke/TIA	291 (2.7)	8809 (2.1)	<.001	5337 (2.9)	3763 (1.6)	<.001	1841 (2.6)	7259 (2.1)	<.001
IBS	213 (2.0)	8103 (2.0)	1.00	3859 (2.1)	4457 (1.9)	<.001	1859 (2.6)	6457 (1.9)	<.001
IBD	119 (1.1)	7239 (1.8)	<.001	3212 (1.7)	4146 (1.8)	.37	1138 (1.6)	6220 (1.8)	<.001
Alcohol use disorder	196 (1.8)	5205 (1.3)	<.001	3170 (1.7)	2231 (0.9)	<.001	1085 (1.5)	4316 (1.2)	<.001
Single-site cancer	135 (1.3)	4905 (1.2)	.59	2523 (1.4)	2517 (1.1)	<.001	830 (1.2)	4210 (1.2)	.22
Chronic heart failure	226 (2.1)	4429 (1.1)	<.001	3342 (1.8)	1313 (0.6)	<.001	1199 (1.7)	3456 (1.0)	<.001
Atrial fibrillation	183 (1.7)	4070 (1.0)	<.001	2673 (1.4)	1580 (0.7)	<.001	834 (1.2)	3419 (1.0)	<.001
Epilepsy	156 (1.4)	3196 (0.8)	<.001	1486 (0.8)	1866 (0.8)	.85	678 (0.9)	2674 (0.8)	<.001
Acute myocardial infarction	116 (1.1)	3057 (0.7)	<.001	2482 (1.3)	691 (0.3)	<.001	660 (0.9)	2513 (0.7)	<.001
Multiple sclerosis	72 (0.7)	2116 (0.5)	.03	925 (0.5)	1263 (0.5)	.07	447 (0.6)	1741 (0.5)	<.001
Schizophrenia	91 (0.8)	2083 (0.5)	<.001	1427 (0.8)	747 (0.3)	<.001	532 (0.7)	1642 (0.5)	<.001
Severe constipation	51 (0.5)	1829 (0.4)	.68	973 (0.5)	907 (0.4)	<.001	398 (0.6)	1482 (0.4)	<.001
Psoriasis	56 (0.5)	1746 (0.4)	.14	999 (0.5)	803 (0.3)	<.001	440 (0.6)	1362 (0.4)	<.001
PAD	60 (0.6)	1127 (0.3)	<.001	814 (0.4)	373 (0.2)	<.001	215 (0.3)	972 (0.3)	.36
Metastatic cancer	30 (0.3)	944 (0.2)	.30	504 (0.3)	470 (0.2)	<.001	189 (0.3)	785 (0.2)	.06
Parkinson disease	20 (0.2)	642 (0.2)	.45	358 (0.2)	304 (0.1)	<.001	119 (0.2)	543 (0.2)	.56
Peptic ulcer disease	20 (0.2)	663 (0.2)	.54	413 (0.2)	270 (0.1)	<.001	154 (0.2)	529 (0.2)	<.001
Lymphoma	17 (0.2)	587 (0.1)	.69	323 (0.2)	281 (0.1)	<.001	102 (0.1)	502 (0.1)	.88
Dementia	15 (0.1)	357 (0.1)	.07	216 (0.1)	156 (0.1)	<.001	57 (0.1)	315 (0.1)	.36
Cirrhosis	9 (0.1)	192 (0.0)	.09	158 (0.1)	43 (0.0)	<.001	48 (0.1)	153 (0.0)	.01
Chronic hepatitis B	4 (0.0)	153 (0.0)	.99	80 (0.0)	77 (0.0)	.09	14 (0.0)	143 (0.0)	.006

Abbreviations: CRP, C-reactive protein; HDL-C, high-density lipoprotein cholesterol; IBD, inflammatory bowel disease; IBS, irritable bowel syndrome; IQR, interquartile range; PAD, peripheral artery disease; TIA, transient ischemic attack.

SI conversion factors: To convert CRP to nanomoles per liter, multiply by 9.524; to convert HDL-C to millimoles per liter, multiply by 0.0259; to convert glucose to millimoles per liter, multiply by 0.0555; and to convert triglycerides to millimoles per liter, multiply by 0.0113.

^a^ Inflammation was defined as all measures of CRP level (highly sensitive or not) greater than 10 mg/L over a period of longer than 1 year for a minimum of 2 measures.

^b^ Surrogate insulin resistance was defined as at least 2 of the following: fasting glucose level of 108 mg/dL or higher, HDL-C level of 39 mg/dL or lower, or triglyceride level of 142 mg/dL or higher at baseline.

^c^ Severe obesity was defined by a procedure-fee modifier for a body mass index (calculated as weight in kilograms divided by height in meters squared) of 35 or higher before January 1, 2017, and 40 or higher after January 1, 2017.

Compared with participants with inflammation or severe obesity, participants with IR were older and more likely to be men. Although these 3 groups had a similar mean number of comorbidities, there were apparent differences in the patterns of comorbidity between groups. Compared with participants with inflammation and severe obesity, participants with IR had the highest prevalence of diabetes (8.4% and 9.6%, respectively, vs 11.5%), stroke or transient ischemic attack (2.7% and 2.6% vs 2.9%), inflammatory bowel disease (1.1% and 1.6% vs 1.7%), single-site cancer (1.3% and 1.2% vs 1.4%), and AMI (1.1% and 0.9% vs 1.3%). Compared with participants with IR and severe obesity, participants with inflammation were both the youngest and the oldest, included proportionally more women, and had more chronic pulmonary disease (8.0% and 9.0%, respectively, vs 9.4%), chronic kidney disease (4.1% and 3.6% vs 4.5%), rheumatoid arthritis (2.4% and 2.3% vs 2.9%), chronic heart failure (1.8% and 1.7% vs 2.1%), alcohol use disorder (1.7% and 1.5% vs 1.8%), atrial fibrillation (1.4% and 1.2% vs 1.7%), epilepsy (0.8% and 0.9% vs 1.4%), multiple sclerosis (0.5% and 0.6% vs 0.7%), and peripheral artery disease (0.4% and 0.3% vs 0.6%). Compared with participants with inflammation and IR, participants with severe obesity also included proportionally more women and had more hypertension (24.1% and 26.4%, respectively, vs 27.4%), chronic pain (20.3% and 22.0% vs 25.6%), depression (13.3% and 11.8% vs 14.9%), hypothyroidism (6.6% and 6.5% vs 7.7%), asthma (4.2% and 2.8% vs 4.3%), irritable bowel syndrome (2.0% and 2.1% vs 2.6%), severe constipation (0.5% and 0.5% vs 0.6%), and psoriasis (0.5% and 0.5% vs 0.6%). The demographic and clinical characteristics of the 8 mutually exclusive groups formed by the presence and absence of inflammation, IR, and severe obesity are described separately in eTable 2 in the Supplement. Of note, in participants with inflammation, median CRP was higher in those without severe obesity than those with severe obesity (20.5 vs 19.1 mg/L; *P* < .001). However in participants without inflammation, the reverse was true (2.6 vs 5.2 mg/L; *P* < .001).

### Models Without 2-Way Interactions

In age-adjusted models (Table 2) without 2-way interactions, inflammation was most strongly associated with all-cause mortality (hazard ratio [HR], 2.48; 95% CI, 2.34-2.64), and male sex and IR were associated with AMI to a similar extent (male sex: HR, 2.31; 95% CI, 2.22-2.40; IR: HR, 2.23; 95% CI, 2.14-2.33). Severe obesity and inflammation were most strongly associated with cancer (severe obesity: HR, 1.30; 95% CI, 1.27-1.34; inflammation: HR, 1.29; 95% CI, 1.22-1.37), and severe obesity was most strongly associated with new pulmonary disease (HR, 1.65; 95% CI, 1.62-1.68).

**Table 2.** Age-Adjusted HRs by Inflammation, Insulin Resistance, Severe Obesity, and Sex

Exposure	HR (95% CI)			
	All-Cause Mortality	AMI	Cancer	New Pulmonary Disease
Events, No. (%)	19 351 (4.6)	12 030 (2.9)	40 837 (9.7)	58 335 (14.8)
Inflammation^a^	2.48 (2.34-2.64)^b^	1.07 (0.96-1.19)	1.29 (1.22-1.37)^b^	1.53 (1.47-1.60)
Insulin resistance^c^	1.42 (1.37-1.46)	2.23 (2.14-2.33)^b^	1.04 (1.02-1.06)	1.27 (1.25-1.30)
Severe obesity^d^	1.17 (1.13-1.21)	1.39 (1.33-1.45)	1.30 (1.27-1.34)^b^	1.65 (1.62-1.68)^b^
Male sex	1.35 (1.31-1.39)	2.31 (2.22-2.40)^b^	1.15 (1.13-1.18)	1.05 (1.03-1.07)

Abbreviations: AMI, acute myocardial infarction; HR, hazard ratio.

^a^ Inflammation was defined as all measures of C-reactive protein (highly sensitive or not) greater than 10 mg/L (to convert to nanomoles per liter, multiply by 9.524) over a period of longer than 1 year for a minimum of 2 measures.

^b^ Exposure with the largest magnitude.

^c^ Surrogate insulin resistance was defined as at least 2 of the following: fasting glucose level of 108 mg/dL or higher (to convert to millimoles per liter, multiply by 0.0555), high-density lipoprotein cholesterol level of 39 mg/dL or lower (to convert to millimoles per liter, multiply by 0.0259), or triglyceride level of 142 mg/dL or higher (to convert to millimoles per liter, multiply by 0.0113) at baseline.

^d^ Severe obesity was defined as a procedure-fee modifier for a body mass index (calculated as weight in kilograms divided by height in meters squared) of 35 or higher before January 1, 2017, or 40 or higher after January 1, 2017.

### Models With 2-Way Interactions

**Figure 1.**
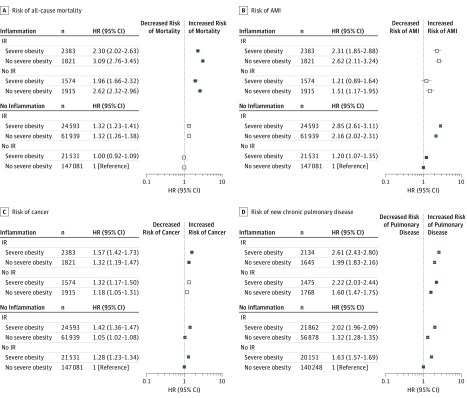
Fully Adjusted Hazard Ratios (HRs) for Clinical Outcomes in Women A-D, The outcomes are all-cause mortality (A), first acute myocardial infarction (AMI) during follow-up (B), first cancer diagnosis (ie, solid tumors [breast, cervical, colorectal, lung, or prostate cancer], lymphoma, and metastatic cancer of any origin) during follow-up (C), and new chronic pulmonary disease (eg, chronic obstructive pulmonary disease, bronchitis, pneumonoconiosis, or asthma) in those without prior chronic pulmonary disease (D). Hollow markers indicate no significant difference between participants with and without severe obesity. IR indicates insulin resistance.

**Figure 2.**
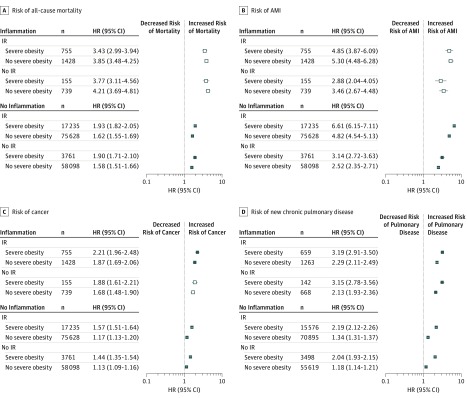
Fully Adjusted Hazard Ratios (HRs) for Clinical Outcomes in Men A-D, The outcomes are all-cause mortality (A), first acute myocardial infarction (AMI) during follow-up (B), first cancer diagnosis (ie, solid tumors [breast, cervical, colorectal, lung, or prostate cancer], lymphoma, and metastatic cancer of any origin) during follow-up (C); and new chronic pulmonary disease (eg, chronic obstructive pulmonary disease, bronchitis, pneumonoconiosis, or asthma) in those without prior chronic pulmonary disease (D). Hollow markers indicate no significant difference between participants with and without severe obesity. IR indicates insulin resistance.

The fully adjusted results were very similar to the age-adjusted analyses (Figure 1 and Figure 2; eTable 3 in the Supplement). However, results in the fully adjusted model were slightly attenuated compared with those from the age-adjusted model.

#### Women

In women (Figure 1), inflammation and IR were associated with increases in risk of all outcomes. Compared with IR, inflammation was associated with a higher risk of all-cause mortality (HR, 1.71; 95% CI, 1.51-1.94) and new pulmonary disease (HR, 1.14; 95% CI, 1.05-1.23), regardless of severe obesity (eTable 4 in Supplement). However, IR was associated with a higher risk of AMI than inflammation in women (HR, 1.81; 95% CI, 1.41-2.33). The association of IR with risk of cancer was similar to the association of inflammation with cancer (HR, 1.03; 95% CI, 0.93-1.15).

Unlike inflammation and IR, the presence of severe obesity was not always associated with increased risk. The risk associated with severe obesity within strata of chronic inflammation and IR is shown in eTable 4 in the Supplement. For women with inflammation, the association of severe obesity with each outcome was similar among women with and without IR.

In women with inflammation, regardless of IR status, severe obesity was associated with a higher risk of new pulmonary disease (HR, 1.34; 95% CI, 1.23-1.46) and cancer (HR, 1.16; 95% CI, 1.03-1.30) compared with the absence of severe obesity. In contrast, among this group, severe obesity was associated with a lower risk of mortality (HR, 0.75; 95% CI, 0.65-0.86) and no evidence of excess risk of AMI (HR, 0.85; 95% CI, 0.67-1.07) compared with the absence of severe obesity.

Among women without inflammation, the association of severe obesity with each outcome was again similar among women with and without IR. In women without inflammation, regardless of IR status, severe obesity was associated with higher risk of new pulmonary disease (HR, 1.58; 95% CI, 1.54-1.62), cancer (HR, 1.32; 95% CI, 1.28-1.36), and AMI (HR, 1.26; 95% CI, 1.17-1.36) but not of all-cause mortality (HR, 1.00, 95% CI, 0.95-1.06) compared with women without severe obesity.

In contrast, for women with IR, the association of obesity with each outcome was different among women with and without inflammation. For example, among women with IR, severe obesity was associated with higher risk of AMI when inflammation was absent (HR, 1.32; 95% CI, 1.22-1.42) but not when inflammation was present (HR, 0.88; 95% CI, 0.69-1.11). Similar differences in the association of severe obesity with all-cause mortality among women with IR were observed in strata defined by the presence and absence of inflammation.

#### Men

In men, inflammation and IR were likewise associated with increases in risk of all outcomes. Compared with IR, inflammation was associated with a higher risk of all-cause mortality (HR, 2.39; 95% CI, 2.10-2.73), new pulmonary disease (HR, 1.54; 95% CI, 1.39-1.70), and cancer (HR, 1.37; 95% CI, 1.21-1.55), regardless of severe obesity (eTable 4 in Supplement). However, the reverse was true for AMI, where IR in men was associated with higher risk (HR, 1.60, 95% CI, 1.24-2.06) compared with inflammation. Similar to the results for women, the presence of severe obesity was not always associated with increased risk.

For men with inflammation, the association of severe obesity with each outcome was similar among men with and without IR. In men with inflammation, regardless of IR status, severe obesity was associated with a higher risk of new pulmonary disease (HR, 1.41; 95% CI, 1.29-1.54) and cancer (HR, 1.17; 95% CI, 1.04-1.32) compared with men with no severe obesity, but no significant association of severe obesity with the risks of mortality (HR, 0.89; 95% CI, 0.78-1.02) or AMI (HR, 0.90, 95% CI, 0.71-1.14) was found.

Among men without inflammation, the association of severe obesity with each outcome was again similar among men with and without IR. In men without inflammation, regardless of IR status, severe obesity was associated with a higher risk of all 4 outcomes (new pulmonary disease: HR, 1.65; 95% CI, 1.60-1.71; AMI: HR, 1.35; 95% CI, 1.27-1.43; cancer: HR, 1.34; 95% CI, 1.28-1.39; and all-cause mortality: HR, 1.20, 95% CI, 1.13-1.26) than in men without severe obesity.

In contrast, for men with IR, the association of obesity with each outcome was different among men with and without inflammation. For example, among men with IR, severe obesity was associated with higher risk of AMI when inflammation was absent (HR, 1.37; 95% CI, 1.29-1.45) but not when inflammation was present (HR, 0.91; 95% CI, 0.72-1.15). Similar differences in the association of severe obesity with all-cause mortality among men with IR were observed in strata defined by the presence and absence of inflammation. In sensitivity analyses, our alternative definition for IR did not change the results in any critical way (eTable 5 in the Supplement).

Men were at a significantly heightened risk compared with women in all 8 permutations of inflammation, IR, and severe obesity for all outcomes (eTable 4 in Supplement) with 1 exception: among participants with IR but without inflammation or severe obesity, the associated risk of new pulmonary disease was similar for women and men (HR, 1.02; 95% CI, 0.99-1.04).

## Discussion

When considered individually in participants of both sexes, severe obesity, evidence of systemic inflammation, and markers of IR were all associated with excess risk of adverse outcomes. Stratification on sex and consideration of the joint associations of the different exposures with outcomes revealed a more complex picture. In particular, the presence of severe obesity was not always associated with increased risk, especially in women. Specifically, we found that in women and men with evidence of inflammation, severe obesity was associated with increased risks of incident pulmonary disease and cancer but not with an excess risk of all-cause mortality or incident AMI. In fact, in women with inflammation, severe obesity was associated with a lower risk of death than in women without severe obesity. Similar to chronic inflammation, severe obesity was not always associated with higher risk in participants with IR; however, compared with IR, the presence or absence of inflammation was a greater modifier of associated risk.

Potential mechanisms behind the higher risk of adverse outcomes, such as death, AMI, cancer, and lung disease, in people with obesity have been discussed elsewhere. Why might severe obesity not be associated with excess risk of mortality and AMI in people with systemic inflammation? It is known that in chronic inflammation, adenosine triphosphate is driven out of cells,^39^ which ultimately results in increased fat production and decreased energy.^40,41^ Some have hypothesized that slower metabolic processes prevent cell death and organ injury in acute conditions such as sepsis.^42^ We speculate that a similar protective phenomenon might operate in chronic inflammation, which could increase the propensity of individuals with chronic inflammation to also have obesity. If true, the simultaneous presence of obesity and chronic inflammation might identify people who are better equipped to withstand catabolic processes in chronic diseases. Since inflammation attenuates progenitor cell function and survival,^43^ and endothelial progenitor cells have been reported to be positively associated with markers of adiposity,^44^ this may be another reason why obesity may be of benefit in chronic inflammatory conditions.

It is well recognized that clinical populations with heart disease, kidney disease, and cancer are actually collections of diseases with a similar phenotype but potentially different etiologies, pathophysiologies, prognoses, and treatments. In our study, participants with IR largely had low-grade elevations of CRP levels (median [IQR] 3.7 [1.8-8.7] mg/L); few had chronic inflammation as we defined it. Specifically, we found that only 3.4% of participants with IR had all CRP levels of more than 10 mg/L, suggesting that IR and inflammation (as we defined them) identify different populations. While obesity is often considered to represent a single phenotype, it is advanced potentially by a variety of pathways.^3^ In our study, among participants with or without inflammation, severe obesity conferred different prognoses, suggesting that obesity should be characterized as a syndrome rather than a single disease.

In a retrospective cohort of patients receiving hemodialysis,^45^ we found that higher quintiles of BMI were associated with longer life expectancy but only in the presence of elevated chronic inflammation. In an editorial by Drechsler and Wanner, they advised “resolving the inflammation and treating the underlying causes”^46^ rather than prescribing weight loss (an intervention proven to be unattainable^47^ in the long term except through bariatric surgery). Similarly, synthesizing experimental data from metabolic studies, Wlodek and Gonzales concluded that “obesity is caused by the metabolic mechanisms of defense.”^41^

Alternatively, certain forms of obesity may occur together with causes of systemic inflammation that are not in themselves immediately harmful, such as small intestinal bacterial overgrowth.^5,48,49^ These unmeasured conditions that are associated with obesity and inflammation may confound the association of severe obesity with adverse outcomes. Also, given that outcomes differed by sex in our study, these potential confounders may occur more frequently in women.

### Limitations

Our study has several important limitations that should be considered when interpreting results. Only 9.3% of the general population in Alberta had the required blood work results and a medical procedure used to define our 8 exposure groups. Thus, our study population was highly selected and had more comorbidity than in the excluded population (eTable 1 in the Supplement). We used a crude definition of obesity, and therefore, some participants would have been incorrectly classified as never having severe obesity. Also, BMI is an imperfect measure of adiposity as assessed by other measures of body composition, such as dual-energy x-ray absorptiometry, magnetic resonance imaging, and computed tomography, which would delineate sarcopenic obesity from other forms of obesity. Furthermore, BMI does not account for body shape or waist-to-hip ratio. Only Alberta residents who had 2 values of CRP over a period longer than 1 year and at least 1 measure of fasting glucose, HDL-C, and triglyceride cholesterol levels were included in the study. While fasting glucose and cholesterol levels are used as a general screening tool, assessing CRP is not a standard of care. Because data from a reference measure of IR (eg, hyperinsulinemic euglycemic clamp or fasting insulin levels) were not available, we used commonly measured parameters (fasting glucose, fasting HDL-C, and triglyceride levels) to define IR rather than a reference standard or indices, such as the Homeostatic Model Assessment of Insulin Resistance or the Quantitative Insulin Sensitivity Check Index.^50^ Our surrogates for IR measure hepatic IR and not peripheral IR, and thus, some participants will have been misclassified as not having IR when they do. Thus, whether our findings are generalizable to a broader population is uncertain. Further, although we adjusted for a panel of more than 30 potential comorbidities, we did not have data on behaviors such as tobacco use, physical activity, dietary habits (including the quality of nutrients, such as the fructose, purine, and antioxidant content^51^), or experiences of weight-related discrimination^52^; thus, residual confounding by these characteristics is possible. On the other hand, the Western dietary pattern plays a significant role in IR and obesity and thus may also confound the results.

Given the limitations of administrative data, it would be useful to confirm our findings using a prospective longitudinal cohort design where all exposures (ie, fat mass, fasting insulin and glucose levels, and CRP levels) could be assessed in greater detail at baseline and in follow-up along with important clinical outcomes. Future research should also confirm the temporal pattern in which inflammation, IR, and obesity typically occur in humans.

## Conclusions

In conclusion, this study highlights that severe obesity with and without systemic inflammation have different prognoses and that these prognoses are further modified by sex. Our study suggests that populations of people with severe obesity also exhibit clinical heterogeneity, especially in terms of prognosis.

## Notes

Supplement. **eTable 1. **Demographic and Clinical Characteristics by Cohort Inclusion/Exclusion **eTable 2.** Demographic and Clinical Characteristics by Permutations of Inflammation, Insulin Resistance, and Severe Obesity **eTable 3. **Age-Adjusted Hazard Ratios by Inflammation, Insulin Resistance, and Severe Obesity in Women and Men **eTable 4. **Fully Adjusted Hazard Ratios Derivable from Data in Figure 1 and Figure 2 **eTable 5. **Fully Adjusted Hazard Ratios by Inflammation, Insulin Resistance, and Severe Obesity in Women and Men, Where Insulin Resistance is Defined Using the Metabolic Index **eFigure. **Participant Flow Diagram
