# Supplementary material for: Associations of Chronic Inflammation, Insulin Resistance, and Severe Obesity With Mortality, Myocardial Infarction, Cancer, and Chronic Pulmonary Disease
Source: JAMA Netw Open. 2019 Aug 30;2(8):e1910456. doi: 10.1001/jamanetworkopen.2019.10456 (PMC6724168; doi:10.1001/jamanetworkopen.2019.10456)
Supplement: Supplement. — eTable 1. Demographic and Clinical Characteristics by Cohort Inclusion/Exclusion eTable 2. Demographic and Clinical Characteristics by Permutations of Inflammation, Insulin Resistance, and Severe Obesity eTable 3. Age-Adjusted Hazard Ratios by Inflammation, Insulin Resistance, and Severe Obesity in Women and Men eTable 4. Fully Adjusted Hazard Ratios Derivable from Data in Figure 1 and Figure 2 eTable 5. Fully Adjusted Hazard Ratios by Inflammation, Insulin Resistance, and Severe Obesity in Women and Men, Where Insulin Resistance is Defined Using the Metabolic Index eFigure. Participant Flow Diagram [file jamanetwopen-2-e1910456-s001.pdf]

Supplementary Online Content

Wiebe N, Stenvinkel P, Tonelli M. Associations of chronic inflammation, insulin resistance, and severe obesity with mortality, myocardial infarction, cancer, and chronic pulmonary disease. *JAMA Netw Open*. 2019;2(8):e1910456. doi:10.1001/jamanetworkopen.2019.10456

eTable 1. Demographic and Clinical Characteristics by Cohort Inclusion/Exclusion

eTable 2. Demographic and Clinical Characteristics by Permutations of Inflammation, Insulin Resistance, and Severe Obesity

eTable 3. Age-Adjusted Hazard Ratios by Inflammation, Insulin Resistance, and Severe Obesity in Women and Men

eTable 4. Fully Adjusted Hazard Ratios Derivable from Data in Figure 1 and Figure 2

eTable 5. Fully Adjusted Hazard Ratios by Inflammation, Insulin Resistance, and Severe Obesity in Women and Men, Where Insulin Resistance is Defined Using the Metabolic Index

eFigure. Participant Flow Diagram

This supplementary material has been provided by the authors to give readers additional information about their work.

© 2019 Wiebe N et al. *JAMA Network Open.*

eTable 1. Demographic and Clinical Characteristics by Cohort Inclusion/Exclusion

Characteristics	Inclusion	Exclusion	P
N	420,636	4,089,586	
Age, median (IQR), y	45 (34,56)	32 (21,47)	<0.001
18-39	148,667 (35.3)	2,586,565 (63.2)	
40-64	221,929 (52.8)	1,164,472 (28.5)	
65-79	46,646 (11.1)	242,360 (5.9)	
≥80	3,394 (0.8)	96,189 (2.4)	
Women	262,837 (62.5)	1,947,021 (47.6)	<0.001
Social assistance	12,235 (2.9)	107,177 (2.6)	<0.001
Number of morbidities, median (IQR)	0 (0,1)	0 (0,0)	<0.001
Chronic pain	84,281 (20.0)	277,236 (6.8)	<0.001
Hypertension	75,888 (18.0)	348,581 (8.5)	<0.001
Depression	47,073 (11.2)	173,558 (4.2)	<0.001
Chronic pulmonary disease	26,155 (6.2)	120,730 (3.0)	<0.001
Hypothyroidism	25,405 (6.0)	89,462 (2.2)	<0.001
Diabetes	21,736 (5.2)	108,792 (2.7)	<0.001
Chronic kidney disease	10,891 (2.6)	55,943 (1.4)	<0.001
Rheumatoid arthritis	9,857 (2.3)	15,093 (0.4)	<0.001
Asthma	9,556 (2.3)	34,675 (0.8)	<0.001
Stroke/TIA	9,100 (2.2)	53,550 (1.3)	<0.001
IBS	8,316 (2.0)	18,614 (0.5)	<0.001
IBD	7,358 (1.7)	8,347 (0.2)	<0.001
Alcohol use disorder	5,401 (1.3)	38,088 (0.9)	<0.001
Single-site cancer	5,040 (1.2)	26,896 (0.7)	<0.001
Chronic heart failure	4,655 (1.1)	45,370 (1.1)	0.87
Atrial fibrillation	4,253 (1.0)	33,735 (0.8)	<0.001
Epilepsy	3,352 (0.8)	19,630 (0.5)	<0.001
Acute myocardial infarction	3,173 (0.8)	19,500 (0.5)	<0.001
Multiple sclerosis	2,188 (0.5)	8,957 (0.2)	<0.001
Schizophrenia	2,174 (0.5)	16,590 (0.4)	<0.001
Severe constipation	1,880 (0.4)	9,564 (0.2)	<0.001
Psoriasis	1,802 (0.4)	5,812 (0.1)	<0.001
PAD	1,187 (0.3)	8,611 (0.2)	<0.001
Metastatic cancer	974 (0.2)	8,484 (0.2)	0.001
Parkinson disease	662 (0.2)	7,510 (0.2)	<0.001
Peptic ulcer disease	683 (0.2)	3,296 (0.1)	<0.001
Lymphoma	604 (0.1)	3,352 (0.1)	<0.001
Dementia	372 (0.1)	19,646 (0.5)	<0.001
Cirrhosis	201 (0.0)	1,656 (0.0)	0.03
Chronic hepatitis B	157 (0.0)	1,012 (0.0)	<0.001

IBD inflammatory bowel disease, IBS irritable bowel syndrome, IQR inter-quartile range, PAD peripheral artery disease, TIA transient ischemic attack
N (%) or median (IQR) as appropriate

© 2019 Wiebe N et al. *JAMA Network Open.*

eTable 2. Demographic and Clinical Characteristics by Permutations of Inflammation, Insulin Resistance, and Severe Obesity

Characteristics	Inflammation and IR		Inflammation only		IR only		Neither		
	Severe obesity	No severe obesity	Severe obesity	No severe obesity	Severe obesity	No severe obesity	Severe obesity	No severe obesity	P
N	3,138 (0.8)	3,249 (0.8)	1,729 (0.4)	2,654 (0.6)	41,828 (9.9)	137,567 (32.7)	25,292 (6.0)	205,179 (48.8)	-
Age, median (IQR), y	44 (33,53)	49 (38,61)	35 (25,47)	44 (31,57)	48 (39,57)	48 (38,59)	41 (28,52)	43 (32,54)	<0.001
18-39	1,215 (38.7)	885 (27.2)	1,030 (59.6)	1,116 (42.0)	11,127 (26.6)	37,580 (27.3)	11,969 (47.3)	83,745 (40.8)	<0.001
40-64	1,696 (54.0)	1,712 (52.7)	614 (35.5)	1,089 (41.0)	26,287 (62.8)	78,442 (57.0)	11,616 (45.9)	100,473 (49.0)	
65-79	225 (7.2)	583 (17.9)	80 (4.6)	381 (14.4)	4,332 (10.4)	20,100 (14.6)	1,665 (6.6)	19,280 (9.4)	
≥80	2 (0.1)	69 (2.1)	5 (0.3)	68 (2.6)	82 (0.2)	1,445 (1.1)	42 (0.2)	1,681 (0.8)	
Women	2,383 (75.9)	1,821 (56.0)	1,574 (91.0)	1,915 (72.2)	24,593 (58.8)	61,939 (45.0)	21,531 (85.1)	147,081 (71.7)	<0.001
Social assistance	191 (6.1)	169 (5.2)	79 (4.6)	118 (4.4)	1,736 (4.2)	4,247 (3.1)	870 (3.4)	4,825 (2.4)	<0.001
Laboratory results, median (IQR)									
CRP level, mg/L	19.5 (15.2,26.1)	21.1 (15.4,30.6)	18.5 (14.9,24.9)	20.0 (15.3,29.1)	5.5 (2.9,10.4)	3.0 (1.4,6.9)	4.7 (2.5,8.9)	2.3 (1.0,4.7)	<0.001
HDL-C level, mg/dL	37 (35,44)	37 (34,46)	50 (44,58)	52 (45,62)	37 (34,44)	38 (35,49)	52 (45,61)	56 (48,67)	<0.001
Glucose level, mg/dL	114 (108,128)	112 (101,124)	90 (85,97)	90 (85,97)	112 (108,124)	110 (95,117)	90 (85,97)	90 (85,95)	<0.001
Triglyceride level, mg/dL	181 (154,230)	179 (152,232)	125 (86,164)	113 (80,158)	187 (158,241)	179 (152,230)	125 (82,165)	101 (68,156)	<0.001
Number of morbidities, median (IQR)	1 (0,2)	1 (0,2)	0 (0,1)	0 (0,1)	1 (0,2)	1 (0,2)	1 (0,1)	0 (0,1)	<0.001
Chronic pain	741 (23.6)	679 (20.9)	328 (19.0)	439 (16.5)	11,485 (27.5)	27,946 (20.3)	5,874 (23.2)	36,789 (17.9)	<0.001
Hypertension	963 (30.7)	961 (29.6)	255 (14.7)	415 (15.6)	14,321 (34.2)	32,800 (23.8)	4,191 (16.6)	21,982 (10.7)	<0.001
Depression	566 (18.0)	406 (12.5)	218 (12.6)	241 (9.1)	6,484 (15.5)	14,529 (10.6)	3,478 (13.8)	21,151 (10.3)	<0.001
Chronic pulmonary disease	345 (11.0)	341 (10.5)	112 (6.5)	218 (8.2)	4,390 (10.5)	9,794 (7.1)	1,643 (6.5)	9,312 (4.5)	<0.001
Hypothyroidism	240 (7.6)	225 (6.9)	104 (6.0)	143 (5.4)	3,344 (8.0)	8,216 (6.0)	1,823 (7.2)	11,310 (5.5)	<0.001
Diabetes	466 (14.9)	430 (13.2)	3 (0.2)	5 (0.2)	6,382 (15.3)	14,053 (10.2)	65 (0.3)	342 (0.2)	<0.001
Chronic kidney disease	156 (5.0)	219 (6.7)	21 (1.2)	84 (3.2)	1,934 (4.6)	5,236 (3.8)	453 (1.8)	2,788 (1.4)	<0.001
Rheumatoid arthritis	60 (1.9)	119 (3.7)	30 (1.7)	106 (4.0)	979 (2.3)	3,321 (2.4)	614 (2.4)	4,628 (2.3)	<0.001
Asthma	201 (6.4)	115 (3.5)	60 (3.5)	72 (2.7)	1,870 (4.5)	2,983 (2.2)	960 (3.8)	3,295 (1.6)	<0.001
Stroke/TIA	76 (2.4)	133 (4.1)	19 (1.1)	63 (2.4)	1,298 (3.1)	3,830 (2.8)	448 (1.8)	3,233 (1.6)	<0.001
IBS	82 (2.6)	72 (2.2)	33 (1.9)	26 (1.0)	1,158 (2.8)	2,547 (1.9)	586 (2.3)	3,812 (1.9)	<0.001
IBD	33 (1.1)	39 (1.2)	15 (0.9)	32 (1.2)	690 (1.6)	2,450 (1.8)	400 (1.6)	3,699 (1.8)	<0.001
Alcohol use disorder	54 (1.7)	83 (2.6)	18 (1.0)	41 (1.5)	764 (1.8)	2,269 (1.6)	249 (1.0)	1,923 (0.9)	<0.001
Single-site cancer	27 (0.9)	58 (1.8)	15 (0.9)	35 (1.3)	544 (1.3)	1,894 (1.4)	244 (1.0)	2,223 (1.1)	<0.001
Chronic heart failure	68 (2.2)	108 (3.3)	13 (0.8)	37 (1.4)	941 (2.2)	2,225 (1.6)	177 (0.7)	1,086 (0.5)	<0.001
Atrial fibrillation	47 (1.5)	83 (2.6)	15 (0.9)	38 (1.4)	598 (1.4)	1,945 (1.4)	174 (0.7)	1,353 (0.7)	<0.001
Epilepsy	33 (1.1)	44 (1.4)	30 (1.7)	49 (1.8)	390 (0.9)	1,019 (0.7)	225 (0.9)	1,562 (0.8)	<0.001
Acute myocardial infarction	31 (1.0)	65 (2.0)	0 (0.0)	20 (0.8)	563 (1.3)	1,823 (1.3)	66 (0.3)	605 (0.3)	<0.001
Multiple sclerosis	29 (0.9)	14 (0.4)	13 (0.8)	16 (0.6)	255 (0.6)	627 (0.5)	150 (0.6)	1,084 (0.5)	<0.001
Schizophrenia	31 (1.0)	42 (1.3)	6 (0.3)	12 (0.5)	410 (1.0)	944 (0.7)	85 (0.3)	644 (0.3)	<0.001
Severe constipation	14 (0.4)	23 (0.7)	5 (0.3)	9 (0.3)	260 (0.6)	676 (0.5)	119 (0.5)	774 (0.4)	<0.001
Psoriasis	18 (0.6)	16 (0.5)	8 (0.5)	14 (0.5)	293 (0.7)	672 (0.5)	121 (0.5)	660 (0.3)	<0.001
PAD	10 (0.3)	34 (1.0)	2 (0.1)	14 (0.5)	171 (0.4)	599 (0.4)	32 (0.1)	325 (0.2)	<0.001

© 2019 Wiebe N et al. *JAMA Network Open.*

Characteristics	Inflammation and IR		Inflammation only		IR only		Neither		
	Severe obesity	No severe obesity	Severe obesity	No severe obesity	Severe obesity	No severe obesity	Severe obesity	No severe obesity	P
Metastatic cancer	12 (0.4)	9 (0.3)	3 (0.2)	6 (0.2)	118 (0.3)	365 (0.3)	56 (0.2)	405 (0.2)	0.001
Parkinson disease	4 (0.1)	13 (0.4)	0 (0.0)	3 (0.1)	83 (0.2)	258 (0.2)	32 (0.1)	269 (0.1)	<0.001
Peptic ulcer disease	7 (0.2)	5 (0.2)	4 (0.2)	4 (0.2)	110 (0.3)	291 (0.2)	33 (0.1)	229 (0.1)	<0.001
Lymphoma	3 (0.1)	7 (0.2)	3 (0.2)	4 (0.2)	70 (0.2)	243 (0.2)	26 (0.1)	248 (0.1)	0.001
Dementia	3 (0.1)	9 (0.3)	0 (0.0)	3 (0.1)	37 (0.1)	167 (0.1)	17 (0.1)	136 (0.1)	<0.001
Cirrhosis	4 (0.1)	4 (0.1)	0 (0.0)	1 (0.0)	37 (0.1)	113 (0.1)	7 (0.0)	35 (0.0)	<0.001
Chronic hepatitis B	0 (0.0)	2 (0.1)	1 (0.1)	1 (0.0)	12 (0.0)	66 (0.0)	1 (0.0)	74 (0.0)	0.04

CRP C-reactive protein, HDL-C high-density lipoprotein cholesterol, IBD inflammatory bowel disease, IBS irritable bowel syndrome, IQR inter-quartile range, IR insulin resistance, PAD peripheral artery disease, TIA transient ischemic attack

N (%) or median (IQR) as appropriate

Inflammation is defined as all measures of C-reactive protein (highly sensitive or not) above 10 mg/L (>95 nmol/L) over a period of longer than 1 year for a minimum of 2 measures. Surrogate IR is defined as at least 2 of the following: fasting glucose level of 108 mg/dL or higher (≥6.0 mmol/L), HDL-C level of 39 mg/dL or lower (≤1.0 mmol/L), or triglyceride level of 142 mg/dL or higher (≥1.6 mmol/L) at baseline. Severe obesity is defined as a procedure-fee modifier for a body mass index of 35 kg/m^2 or higher before January 1, 2017, or 40 kg/m^2 or higher after January 1, 2017.

© 2019 Wiebe N et al. *JAMA Network Open.*

eTable 3. Age-Adjusted Hazard Ratios by Inflammation, Insulin Resistance, and Severe Obesity in Women and Men

Groups	All-cause mortality	AMI	Cancer	New Pulmonary
Events, n (%)	9,952 (3.8)	4,376 (1.7)	23,495 (8.9)	34,260 (13.9)
**Women**				
Inflammation and IR				
Severe obesity	3.32 (2.92,3.79)	2.69 (2.15,3.35)	1.54 (1.39,1.70)	2.86 (2.66,3.08)
No severe obesity	4.14 (3.70,4.63)	2.96 (2.39,3.67)	1.32 (1.18,1.47)	2.19 (2.01,2.37)
Inflammation only				
Severe obesity	2.24 (1.89,2.65)	1.27 (0.94,1.73)	1.33 (1.17,1.51)	2.31 (2.11,2.53)
No severe obesity	2.81 (2.49,3.18)	1.55 (1.20,2.00)	1.19 (1.06,1.33)	1.67 (1.53,1.82)
IR only				
Severe obesity	1.77 (1.66,1.88)	3.30 (3.04,3.60)	1.39 (1.33,1.44)	2.22 (2.15,2.29)
No severe obesity	1.61 (1.55,1.68)	2.37 (2.21,2.53)	1.04 (1.01,1.07)	1.38 (1.35,1.42)
Neither				
Severe obesity	1.09 (1.00,1.18)	1.26 (1.12,1.42)	1.28 (1.22,1.33)	1.70 (1.65,1.76)
No severe obesity	1.00	1.00	1.00	1.00
**Men**				
Events, n (%)	9,399 (6.0)	7,654 (4.9)	17,342 (11.0)	24,075 (16.2)
Inflammation and IR				
Severe obesity	5.02 (4.37,5.76)	5.75 (4.59,7.21)	2.25 (2.00,2.52)	3.32 (3.03,3.64)
No severe obesity	5.16 (4.68,5.69)	6.06 (5.12,7.16)	1.92 (1.74,2.12)	2.42 (2.23,2.63)
Inflammation only				
Severe obesity	4.49 (3.71,5.43)	3.07 (2.18,4.32)	1.96 (1.67,2.30)	3.17 (2.80,3.59)
No severe obesity	4.65 (4.08,5.30)	3.58 (2.77,4.63)	1.75 (1.55,1.99)	2.18 (1.97,2.42)
IR only				
Severe obesity	2.50 (2.36,2.65)	7.60 (7.09,8.16)	1.58 (1.52,1.64)	2.24 (2.17,2.32)
No severe obesity	1.89 (1.81,1.97)	5.20 (4.90,5.53)	1.18 (1.15,1.22)	1.33 (1.30,1.36)
Neither				
Severe obesity	2.05 (1.85,2.26)	3.27 (2.83,3.77)	1.47 (1.38,1.57)	2.04 (1.93,2.14)
No severe obesity	1.55 (1.48,1.63)	2.48 (2.31,2.66)	1.15 (1.12,1.19)	1.14 (1.11,1.17)

AMI acute myocardial infarction, HDL-C high-density lipoprotein cholesterol, IR insulin resistance

© 2019 Wiebe N et al. *JAMA Network Open.*

Inflammation is defined as all measures of C-reactive protein (highly sensitive or not) above 10 mg/L (>95 nmol/L) over a period of longer than 1 year for a minimum of 2 measures. Surrogate IR is defined as at least 2 of the following: fasting glucose level of 108 mg/dL or higher (≥6.0 mmol/L), HDL-C level of 39 mg/dL or lower (≤1.0 mmol/L), or triglyceride level of 142 mg/dL or higher (≥1.6 mmol/L) at baseline. Severe obesity is defined as a procedure-fee modifier for a body mass index of 35 kg/m^2 or higher before January 1, 2017, or 40 kg/m^2 or higher after January 1, 2017.

Clinical outcomes were all-cause death, first acute myocardial infarction during follow-up, first cancer diagnosis during follow-up, and new chronic pulmonary disease (chronic obstructive pulmonary disease, bronchitis, pneumoconiosis, asthma, etc; in those without prior chronic pulmonary disease). The 26,155 (6.2%) participants with chronic pulmonary disease at baseline were excluded from this analysis. Cancers included solid tumors (breast, cervical, colorectal, lung, or prostate cancer), lymphoma, and metastatic cancer of any origin.

There is one model for each outcome. Besides adjustment for these three exposures, sex and their six two-way interactions, the models are adjusted for age (18-39, 40-64, 65-79, ≥80 years).

Hazard ratios with 95% confidence intervals are presented. Dark gray shading indicates that no severe obesity is significantly favoured (P<0.05) over severe obesity. Light gray shading indicates that risks are equivocal and no shading indicates severe obesity is significantly favoured over no severe obesity.

© 2019 Wiebe N et al. *JAMA Network Open.*

eTable 4. Fully Adjusted Hazard Ratios Derivable from Data in Figure 1 and Figure 2

Groups	Comparison	All-cause mortality	AMI	Cancer	New pulmonary disease
Women					
Inflammation and IR	Severe obesity vs no severe obesity	0.74 (0.65,0.86)	0.88 (0.69,1.11)	1.18 (1.05,1.33)	1.31 (1.20,1.43)
Inflammation only	Severe obesity vs no severe obesity	0.75 (0.64,0.87)	0.80 (0.62,1.03)	1.13 (1.00,1.27)	1.39 (1.27,1.52)
IR only	Severe obesity vs no severe obesity	1.00 (0.94,1.06)	1.32 (1.22,1.42)	1.35 (1.30,1.41)	1.54 (1.49,1.59)
Neither	Severe obesity vs no severe obesity	1.00 (0.93,1.09)	1.20 (1.07,1.35)	1.28 (1.23,1.34)	1.63 (1.57,1.69)
Inflammation	Severe obesity vs no severe obesity	0.75 (0.65,0.86)	0.85 (0.67,1.07)	1.16 (1.03,1.30)	1.34 (1.23,1.46)
No inflammation	Severe obesity vs no severe obesity	1.00 (0.95,1.06)	1.26 (1.17,1.36)	1.32 (1.28,1.36)	1.58 (1.54,1.62)
All	Inflammation vs IR<br>IR vs inflammation	1.71 (1.51,1.94)	0.55 (0.42,0.71)<br>1.81 (1.41,2.33)	1.03 (0.93,1.15)	1.14 (1.05,1.23)
Men					
Inflammation and IR	Severe obesity vs no severe obesity	0.89 (0.78,1.02)	0.91 (0.72,1.15)	1.18 (1.05,1.33)	1.39 (1.27,1.52)
Inflammation only	Severe obesity vs no severe obesity	0.90 (0.77,1.05)	0.83 (0.64,1.08)	1.12 (0.99,1.27)	1.48 (1.34,1.62)
IR only	Severe obesity vs no severe obesity	1.19 (1.13,1.26)	1.37 (1.29,1.45)	1.35 (1.29,1.40)	1.63 (1.58,1.69)
Neither	Severe obesity vs no severe obesity	1.20 (1.10,1.32)	1.25 (1.10,1.41)	1.28 (1.21,1.36)	1.73 (1.65,1.82)
Inflammation	Severe obesity vs no severe obesity	0.89 (0.78,1.02)	0.90 (0.71,1.14)	1.17 (1.04,1.32)	1.41 (1.29,1.54)
No inflammation	Severe obesity vs no severe obesity	1.20 (1.13,1.26)	1.35 (1.27,1.43)	1.34 (1.28,1.39)	1.65 (1.60,1.71)
All	Inflammation vs IR<br>IR vs inflammation	2.39 (2.10,2.73)	0.63 (0.49,0.81)<br>1.60 (1.24,2.06)	1.37 (1.21,1.55)	1.54 (1.39,1.70)

© 2019 Wiebe N et al. *JAMA Network Open.*

Groups	Comparison	All-cause mortality	AMI	Cancer	New pulmonary disease
Severe obesity					
Inflammation and IR	Men vs women	1.49 (1.31,1.69)	2.11 (1.68,2.64)	1.41 (1.25,1.59)	1.22 (1.12,1.34)
Inflammation only	Men vs women	1.92 (1.67,2.21)	2.38 (1.88,3.02)	1.43 (1.26,1.61)	1.42 (1.28,1.56)
IR only	Men vs women	1.47 (1.37,1.57)	2.32 (2.14,2.51)	1.11 (1.06,1.17)	1.08 (1.04,1.12)
Neither	Men vs women	1.89 (1.74,2.06)	2.62 (2.35,2.92)	1.12 (1.06,1.19)	1.25 (1.20,1.31)
All	Men vs women	1.81 (1.67,1.97)	2.49 (2.22,2.78)	1.20 (1.13,1.28)	1.26 (1.21,1.32)
No severe obesity					
Inflammation and IR	Men vs women	1.25 (1.11,1.41)	2.03 (1.63,2.53)	1.41 (1.26,1.59)	1.15 (1.05,1.26)
Inflammation only	Men vs women	1.61 (1.42,1.82)	2.29 (1.83,2.87)	1.43 (1.27,1.61)	1.33 (1.21,1.46)
IR only	Men vs women	1.23 (1.18,1.28)	2.23 (2.11,2.36)	1.11 (1.08,1.15)	1.02 (0.99,1.04)
Neither	Men vs women	1.58 (1.51,1.66)	2.52 (2.35,2.71)	1.13 (1.09,1.16)	1.18 (1.14,1.21)
All	Men vs women	1.30 (1.20,1.42)	2.14 (1.83,2.50)	1.32 (1.22,1.43)	1.14 (1.07,1.22)

AMI acute myocardial infarction, HDL-C high-density lipoprotein cholesterol, IR insulin resistance

Inflammation is defined as all measures of C-reactive protein (highly sensitive or not) above 10 mg/L (>95 nmol/L) over a period of longer than 1 year for a minimum of 2 measures. Surrogate IR is defined as at least 2 of the following: fasting glucose level of 108 mg/dL or higher (≥6.0 mmol/L), HDL-C level of 39 mg/dL or lower (≤1.0 mmol/L), or triglyceride level of 142 mg/dL or higher (≥1.6 mmol/L) at baseline. Severe obesity is defined as a procedure-fee modifier for a body mass index of 35 kg/m^2 or higher before January 1, 2017, or 40 kg/m^2 or higher after January 1, 2017.

Clinical outcomes were all-cause death, first acute myocardial infarction during follow-up, first cancer diagnosis during follow-up, and new chronic pulmonary disease (chronic obstructive pulmonary disease, bronchitis, pneumoconiosis, asthma, etc; in those without prior chronic pulmonary disease). The 26,155 (6.2%) participants with chronic pulmonary disease at baseline were excluded from this analysis. Cancers included solid tumors (breast, cervical, colorectal, lung, or prostate cancer), lymphoma, and metastatic cancer of any origin.

There is one model for each outcome. Besides adjustment for these three exposures, sex and their six two-way interactions, the models are adjusted for age (18-39, 40-64, 65-79, ≥80 years), social assistance, plus 29 comorbidities: chronic pain, hypertension, depression, chronic pulmonary disease (if not also the outcome), hypothyroidism, diabetes, chronic kidney disease, rheumatoid arthritis, asthma, stroke/transient ischemic attack, irritable bowel syndrome, inflammatory bowel disease, alcohol use disorder, single-site cancer, chronic heart failure, atrial fibrillation, epilepsy, acute myocardial infarction, multiple sclerosis, schizophrenia, severe constipation, psoriasis, peripheral artery disease, metastatic cancer, Parkinson disease, peptic ulcer disease, lymphoma, dementia, cirrhosis, and chronic hepatitis B.

© 2019 Wiebe N et al. *JAMA Network Open.*

Hazard ratios with 95% confidence intervals are presented. Dark gray shading indicates that no severe obesity is significantly favoured (P<0.05) over severe obesity. Light gray shading indicates that risks are equivocal and no shading indicates severe obesity is significantly favoured over no severe obesity.

© 2019 Wiebe N et al. *JAMA Network Open.*

eTable 5. Fully Adjusted Hazard Ratios by Inflammation, Insulin Resistance, and Severe Obesity in Women and Men, where Insulin Resistance Is Defined Using the Metabolic Index

Groups	All-cause mortality	AMI	Cancer	New Pulmonary disease
**Women**				
Events, n (%)	9,952 (3.8)	4,376 (1.7)	23,495 (8.9)	34,260 (13.9)
Inflammation and IR				
Severe obesity	2.40 (2.09,2.75)	2.39 (1.90,2.99)	1.64 (1.48,1.82)	2.66 (2.47,2.87)
No severe obesity	3.06 (2.71,3.46)	3.02 (2.44,3.75)	1.41 (1.26,1.58)	2.02 (1.85,2.20)
Inflammation only				
Severe obesity	1.96 (1.67,2.31)	1.14 (0.85,1.54)	1.28 (1.13,1.44)	2.20 (2.01,2.40)
No severe obesity	2.57 (2.28,2.90)	1.36 (1.05,1.76)	1.14 (1.02,1.27)	1.57 (1.44,1.71)
IR only				
Severe obesity	1.39 (1.30,1.48)	2.87 (2.63,3.14)	1.44 (1.38,1.50)	2.08 (2.01,2.15)
No severe obesity	1.35 (1.29,1.41)	2.30 (2.15,2.46)	1.07 (1.04,1.10)	1.36 (1.33,1.40)
Neither				
Severe obesity	1.00 (0.93,1.08)	1.33 (1.20,1.47)	1.29 (1.24,1.35)	1.63 (1.57,1.68)
No severe obesity	1.00	1.00	1.00	1.00
**Men**				
Events, n (%)	9,399 (6.0)	7,654 (4.9)	17,342 (11.0)	24,075 (16.2)
Inflammation and IR				
Severe obesity	3.49 (3.03,4.03)	4.63 (3.67,5.83)	2.21 (1.96,2.49)	3.25 (2.95,3.57)
No severe obesity	3.79 (3.41,4.20)	5.46 (4.61,6.46)	1.87 (1.69,2.08)	2.28 (2.10,2.48)
Inflammation only				
Severe obesity	3.61 (2.98,4.37)	2.59 (1.84,3.66)	1.86 (1.58,2.18)	3.29 (2.91,3.72)
No severe obesity	4.02 (3.51,4.59)	2.87 (2.20,3.76)	1.64 (1.44,1.86)	2.17 (1.96,2.40)
IR only				
Severe obesity	2.00 (1.88,2.13)	6.43 (5.99,6.90)	1.58 (1.51,1.64)	2.19 (2.12,2.26)
No severe obesity	1.65 (1.58,1.73)	4.80 (4.52,5.09)	1.16 (1.12,1.19)	1.33 (1.29,1.36)
Neither				
Severe obesity	1.82 (1.65,2.01)	3.47 (3.04,3.96)	1.53 (1.44,1.63)	2.10 (1.99,2.21)
No severe obesity	1.55 (1.47,1.62)	2.44 (2.28,2.61)	1.17 (1.13,1.20)	1.19 (1.16,1.23)

AMI acute myocardial infarction, HDL-C high-density lipoprotein cholesterol, IR insulin resistance

© 2019 Wiebe N et al. *JAMA Network Open.*

Inflammation is defined as all measures of C-reactive protein (highly sensitive or not) above 10 mg/L (>95 nmol/L) over a period of longer than 1 year for a minimum of 2 measures. Surrogate IR is defined as at least 2 of the following: fasting glucose level of 108 mg/dL or higher (≥6.0 mmol/L), HDL-C level of 39 mg/dL or lower (≤1.0 mmol/L), or triglyceride level of 142 mg/dL or higher (≥1.6 mmol/L) at baseline. Severe obesity is defined as a procedure-fee modifier for a body mass index of 35 kg/m^2 or higher before January 1, 2017, or 40 kg/m^2 or higher after January 1, 2017.

Clinical outcomes were all-cause death, first acute myocardial infarction during follow-up, first cancer diagnosis during follow-up, and new chronic pulmonary disease (chronic obstructive pulmonary disease, bronchitis, pneumoconiosis, asthma, etc; in those without prior chronic pulmonary disease). The 26,155 (6.2%) participants with chronic pulmonary disease at baseline were excluded from this analysis. Cancers included solid tumors (breast, cervical, colorectal, lung, or prostate cancer), lymphoma, and metastatic cancer of any origin.

There is one model for each outcome. Besides adjustment for these three exposures, sex and their six two-way interactions, the models are adjusted for age (18-39, 40-64, 65-79, ≥80 years), social assistance, plus 29 comorbidities: chronic pain, hypertension, depression, chronic pulmonary disease (if not also the outcome), hypothyroidism, diabetes, chronic kidney disease, rheumatoid arthritis, asthma, stroke/transient ischemic attack, irritable bowel syndrome, inflammatory bowel disease, alcohol use disorder, single-site cancer, chronic heart failure, atrial fibrillation, epilepsy, acute myocardial infarction, multiple sclerosis, schizophrenia, severe constipation, psoriasis, peripheral artery disease, metastatic cancer, Parkinson disease, peptic ulcer disease, lymphoma, dementia, cirrhosis, and chronic hepatitis B.

Hazard ratios with 95% confidence intervals are presented. Dark gray shading indicates that no severe obesity is significantly favoured ($P<0.05$) over severe obesity. Light gray shading indicates that risks are equivocal and no shading indicates severe obesity is significantly favoured over no severe obesity.

In this sensitivity analysis, 25,930 (6.5%) were defined as insulin resistant that were not in the primary dataset; and 25,364 (6.3%) were defined as not insulin resistant that were in the primary dataset.

© 2019 Wiebe N et al. *JAMA Network Open.*

eFigure. Participant Flow Diagram

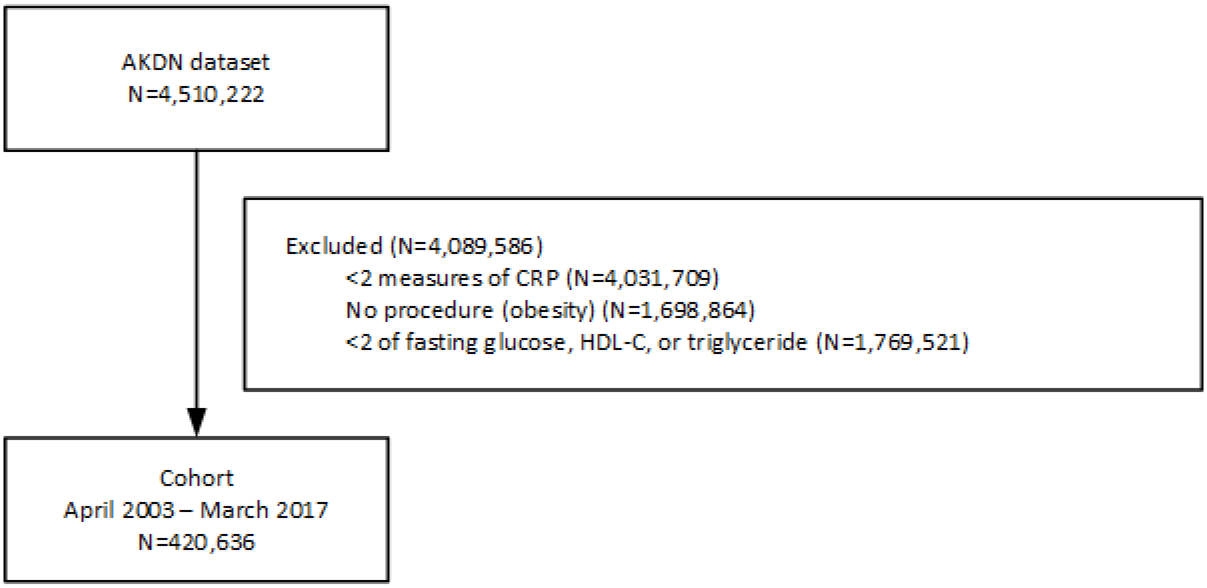

AKDN Alberta Kidney Disease Network, CRP C-reactive protein, HDL-C high-density lipoprotein cholesterol

© 2019 Wiebe N et al. *JAMA Network Open.*
